# Efficient production of recombinant proteins in suspension CHO cells culture using the *Tol2* transposon system coupled with cycloheximide resistance selection

**DOI:** 10.1038/s41598-023-34636-4

**Published:** 2023-05-10

**Authors:** Keina Yamaguchi, Risa Ogawa, Masayoshi Tsukahara, Koichi Kawakami

**Affiliations:** 1 Laboratory of Molecular and Developmental Biology, National Institute of Genetics, Mishima, Shizuoka 411-8540 Japan; 2 Bio Process Research and Development Laboratories, Kyowa Kirin Co., Ltd., 100-1 Hagiwara-machi, Takasaki, Gunma 370-0013 Japan; 3 Department of Genetics, Graduate University for Advanced Studies (SOKENDAI), Mishima, Shizuoka 411-8540 Japan

**Keywords:** Biological techniques, Molecular biology

## Abstract

DNA recombination techniques in mammalian cells has been applied to the production of therapeutic proteins for several decades. To be used for commercial production, established cell lines should stably express target proteins with high productivity and acceptable quality for human use. In the conventional transfection method, the screening process is laborious and time-consuming since superior cell lines had to be selected from an enormous number of transfected cell pools and clonal cell lines with a wide variety of transgene insertion locations. In this study, we demonstrated that the combination of a Tol2 transposon system and cell selection by cycloheximide resistance is an efficient method to express therapeutic proteins, such as human antibody in suspension culture of Chinese hamster ovary cells. The resulting stable cell lines showed constant productivity and cell growth over a long enough cultivation periods for recombinant protein production. We anticipate that this approach will prove widely applicable to protein production in research and development of pharmaceutical products.

## Introduction

DNA recombination techniques in mammalian cells are applied to production of therapeutic proteins for a couple of decades. In many cases, production of recombinant proteins is carried out by generating stable transfected mammalian cell lines expressing the gene of interest (GOI), followed by cultivation in large scale bioreactor. Chinese Hamster Ovary (CHO) cells remain the most popular host cell lines for the production of many therapeutic proteins because of their ability to produce recombinant proteins with human-like posttranslational modifications such as glycosylation or phosphorylation, and also to grow at high cell densities in serum-free suspension culture^1^.

In mammalian cells including CHO cells, however, DNA integration efficiency is generally low using conventional electroporation transfection, and it is not easy to obtain stable transfectants with high levels of protein production since expression of the integrated genes is not always high due to “positional effect”^2^. Thus, the process of screening and isolating highly protein-producing clones has been laborious and time-consuming.

In order to overcome these problems, the gene amplification systems such as the dihydrofolate reductase (dhfr)/methotrexate (MTX) and the glutamine synthetase (GS)/ methionine sulphoximine (MSX) system have been used to increase the copy number of transgenes, resulting in higher levels of GOI expression^3,4^. The chromatin-regulating elements, including insulator elements, matrix/scaffold attachment region (MAR/SAR) elements and ubiquitous chromatin opening elements (UCOEs), have also been used to increase GOI expression levels, independently on the position effects^5^. Recently, genome editing technologies, such as zinc finger nuclease (ZFN), transcription activator-like effector nuclease (TALEN) and clustered regularly interspaced palindromic repeat (CRISPR)/Cas9 systems, have been adapted for biotechnology and medical applications, enabling knock-in of transgenes into any genomic loci that may lead to higher gene expression theoretically^6^.

The Tol2 element is a transposon identified from the Japanese medaka fish, *Oryzias latipes*, that belongs to the hAT family of transposons^7^. It has been shown that the Tol2 element contains a gene for a functional transposase and cis-sequences essential for transposition including the terminal inverted repeats (TIRs) and the subterminal regions (STRs)^8–11^. The two-component transposition system using the Tol2 element has been developed that consists of a donor vector containing the cis-sequences and a transposase-expressing vector. The Tol2 transposition system can create single-copy integrations at multiple loci on the genome efficiently through a “cut-and-paste” mechanism in any vertebrate cells so far tested, including zebrafish and mammalian cells^12–14^. The merit of the Tol2 system is that it has a large-cargo capacity, namely any GOI with the size of even more than 100-kb can be cloned in the transposon-donor vector^15^. The transposon systems, including Tol2, *piggyBac*, and *Sleeping Beauty*, have been applied to production of a recombinant protein, the extracellular domain of the human tumor necrosis factor α receptor 2 (TNFR) fused to a human IgG1 Fc (TNFR:Fc) in CHO cells^16^.

Drug-resistance selection has been commonly used for enrichment and screening of transfected cells. Cycloheximide (CHX), an antibiotic produced by *Streptomyces griseus*, is a potent inhibitor of protein synthesis in most eukaryotes by binding to the 60S ribosomal large subunit, thereby inhibiting the peptidyl elongation reaction on the ribosome and causing cell death. In yeast which is CHX-sensitive, a CHX-resistant mutant was isolated and a gene encoding the ribosomal protein L41 carrying a mutation that conveys CHX-resistant was cloned. The mutant L41 gene was sequenced and the 56th proline to the glutamine mutation was identified^17,18^. Then, to create the CHX-resistant animal cell, Misawa et al.^19^ cloned a gene for the human 60S ribosomal protein L36a, a counterpart of the yeast L41, and introduced a substitution of the 54th glutamine for the proline in the L36a gene. Misawa et al. demonstrated that the mutant L36a gene, when expressed, conferred CHX resistance on adherent CHO cells. It is not clear whether the CHX-resistance selection can be applied to suspension CHO cells to generate stable transfected cell lines in combination with the transposon system.

In this study, we report a new procedure to generate stable transfected cells for recombinant protein production by using suspension CHO cells, combining the Tol2 transposon system with the CHX-resistance selection. We also demonstrate that transfected clonal cell lines show constant productivity and cell growth in long-term culture in the absence of selective pressure.

## Results

### Efficiency of Tol2-mediate gene integration in suspension CHO cells

**Figure 1:**
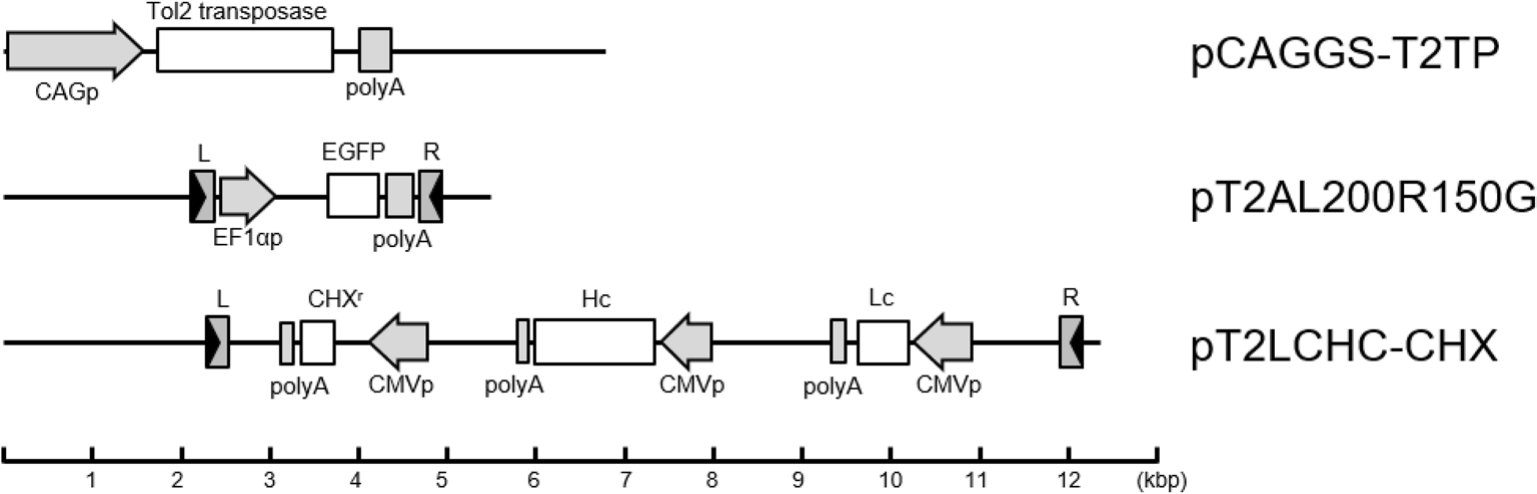
Schematic diagram of transposase-expressing and transposon-donor vectors used in this study. The structures of the transposase-expressing vector pCAGGS-T2TP^13^, the transposon-donor vector pT2AL200R150G^11^ and pT2LCHC-CHX are shown. L: left end of Tol2 cis-sequence, R: right end of Tol2 cis-sequence, CHX^r^: cycloheximide resistance gene, CMVp: cytomegalovirus promoter, Hc: human monoclonal antibody heavy chain gene, Lc: human monoclonal antibody light chain gene.

In order to evaluate the efficiency of gene integration using the Tol2 transposon system (Fig. 1) in suspension CHO cells, we co-transfected the Tol2 donor vector pT2AL200R150G (10 μg) and various amounts of the transposase-expressing vector pCAGGS-T2TP (ranging from 5 to 25 μg) into suspension CHO cells. As a control, cells were transfected with the donor vector pT2AL200R150G alone.

**Figure 2:**
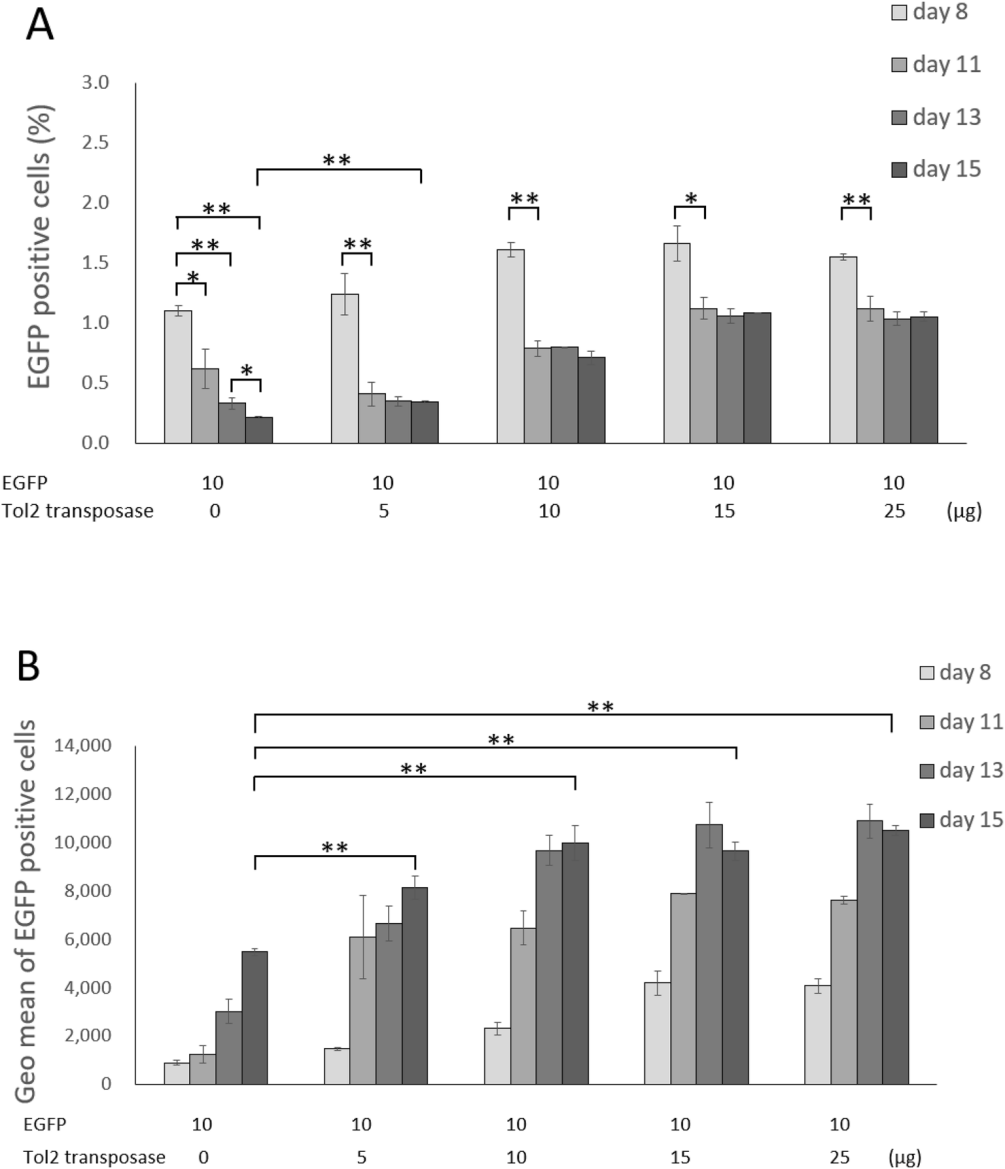
The percentage of EGFP-positive cells and Geometric mean of EGFP expression levels from 8 to 15 days after post-transfection. Cells were co-transfected with pT2AL200R150G (10 μg) and a various amount of the helper vector pCAGGS-T2TP (0–25 μg). In each culture, the percentage of EGFP-positive cells (**A**) and the Geo mean GEFP expression level (**B**) was measured by FACSVerse flow cytometer after 8, 11, 13 and 15 days post-transfection. Data represent mean and standard deviation in triplicate experiments, and asterisks indicate statistical significance determined by student's t test (*P < 0.05, **P < 0.01).

After transfection, cells were cultivated without any selective pressure for 15 days and analyzed for EGFP fluorescence by flow cytometry. For all conditions tested, we observed transient expression of EGFP; namely, the percentages of EGFP-positive cells were 30–35% on one day post-transfection, and then gradually decreased to ~ 1% on the eighth day post-transfection (data not shown). We counted the number of EGFP-positive cells after the eighth day. Without pCAGGS-T2TP, the EGFP-positive cells decreased to 0.2% on the 15th day (Fig. 2A). In contrast, the number of EGFP-positive cells was not decreased to such a low level in cells transfected with pCAGGS-T2TP of 10–25 μg during 11–15 days post transfection (Fig. 2A). Thus, EGFP was stably expressed in cells co-transfected with pCAGGS-T2TP, suggesting stable integration of the transposon-donor construct (Fig. 2A).

We analyzed the fluorescence intensity (Geometric Mean) of EGFP-positive cells that represented the strength of gene expression. On the 15th day post-transfection, cells transfected with 10 μg or more pCAGGS-T2TP resulted in more EGFP-positive cells with stronger EGFP expression than the control transfection without pCAGGS-T2TP (Fig. 2B). For transfection experiments with 15–25 μg of pCAGGS-T2TP, we observed similar levels of EGFP-positive cells with stronger EGFP expression. We think the stronger EGFP expression may be caused by multiple single insertions on the genome created by the Tol2 transposition system. We did not observe any toxic effects on cells transfected with all of these conditions (data not shown). Thus, we decided to perform subsequent experiments with a condition using 25 μg of pCAGGS-T2TP.

### Evaluation of the Tol2 transposition for the CHX-resistance selection

Next, we examined the CHX-resistance selection system combined with the Tol2 transposon system. We constructed pT2LCHC-CHX containing IgG genes (Lc and Hc) for a human monoclonal antibody and CHX-resistant gene driven by the CMV promoters. 10 μg of pT2LCHC-CHX with 25 μg of pCAGGS-T2TP were transfected in suspension CHO cells. As a control, the linearized 10 μg of pT2LCHC-CHX was transfected without pCAGGS-T2TP. After transfection, cells were seeded by a limiting dilution procedure onto 96-well plate followed by cultivation without selective pressure. At 4 days post-transfection, cells were subjected to three different selection conditions (3, 10 and 30 μM CHX) for 3 weeks. After the CHX selection, we observed CHX-resistant colonies in 20/96 wells with 3 μM CHX, 18/96 wells with 10 μM CHX, and 22/96 wells with 30 μM CHX, respectively. Thus, CHX-resistant colonies were obtained under all three conditions at similar frequencies, suggesting CHX expression from stable integrated copies of the CHX gene through the transposon system can confer resistance to fairly high concentrations of CHX. In contrast, any CHX-resistant colonies were not obtained from cells transfected without pCAGGS-T2TP, indicating integration created by the conventional transfection system does not work well with CHX selection.

**Figure 3:**
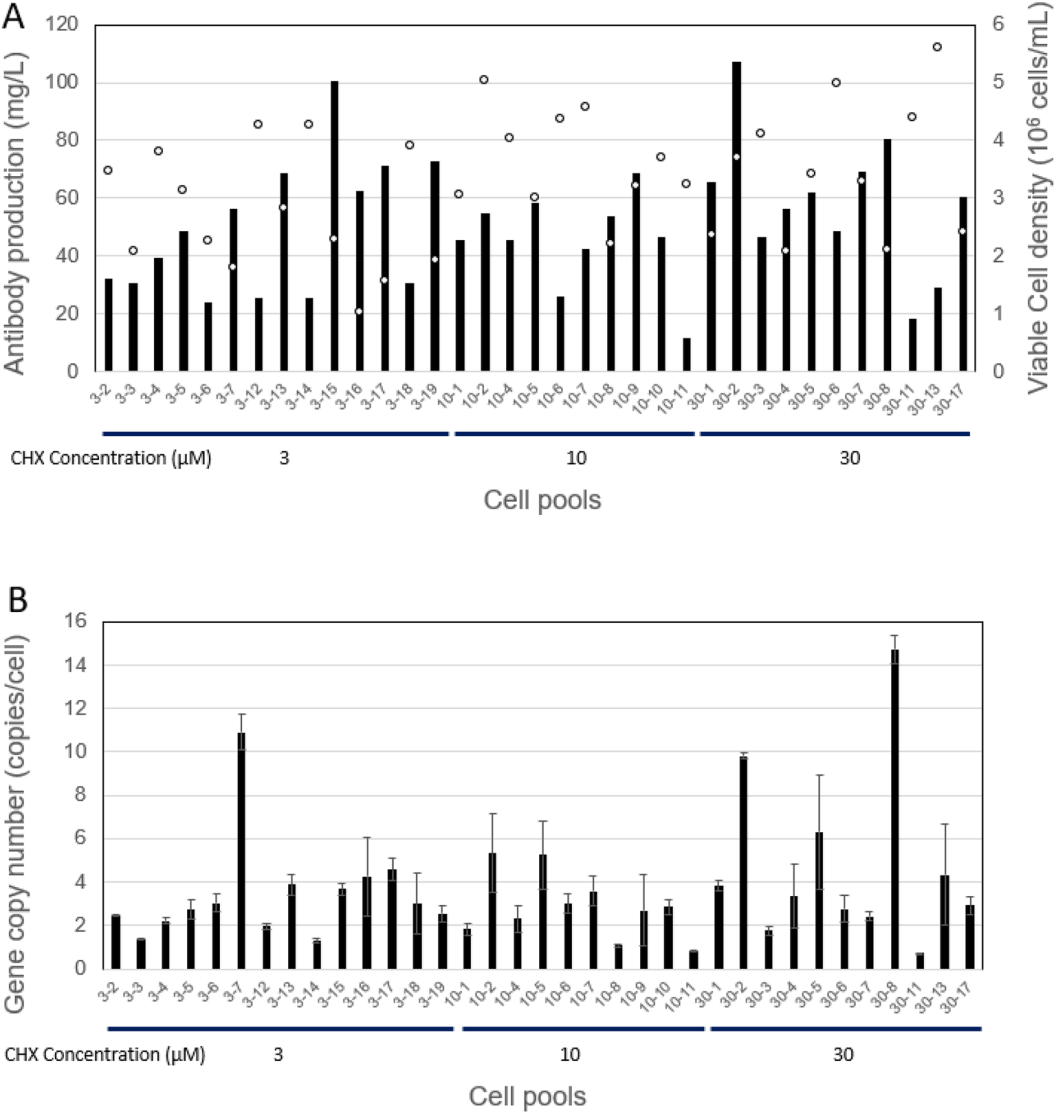
Comparison of the antibody production, cell growth and the integrated gene copy number from each cell pools. Cells were co-transfected with a fixed amount of Tol2 donor vector T2-pLCHC-CHX coding for human monoclonal antibody (10 μg) and the helper vector pCAGGS-T2TP coding for Tol2 transposase (25 μg) and selected in 3, 10 or 30 μM CHX for 3 weeks to generate the cell pools, respectively. (**A**) The antibody production (vertical bars) was measured by protein-A HPLC and viable cell density (open circles) was measured by Vi-CELL XR Cell Viability analyzer at the end of 5 days cultivation. (**B**) The mean and standard deviation in duplication measurement of integrated gene copy number of human monoclonal antibody was measured by qPCR.

The CHX-resistant cells from 14 wells with 3 μM CHX, 10 wells with 10 μM CHX and 11 wells with 30 μM CHX were cultured separately in the presence of CHX of the respective concentration for 5-days. Production of human antibody and viable cell density were measured for cells from each well. Viable cell density varied in the range of 10.3–56.0 × 10^5^ cells/mL. The antibody concentrations in the supernatants were 24.1–100.4 mg/L in 3 μM CHX, 11.5–68.8 mg/L in 10 μM CHX and 18.4–107.3 mg/L in 30 μM CHX (Fig. 3A). We do not see significant correlation between cell density and antibody production. The top two high producer cell lines were obtained from well 30-2 (107.3 mg/L) and 3-15 (100.4 mg/L) with selection conditions of 30 μM CHX and 3 μM CHX, respectively.

Then we measured the gene copy number of integrated pT2LCHC-CHX by qPCR using the genomic DNA extracted from each cell pools (Fig. 3B). We found the copy numbers varied from 1 to 15 in average in these cell pools. Single copy integration could confer resistance to 30 μM CHX (30-11). The top two high producer lines contained 10 copies (30-2) and 4 copies (3-15), respectively. We found no strong correlation between the antibody production and the gene copy number.

### Evaluation of clonal cell lines derived from CHX-resistance cell pools

**Figure 4:**
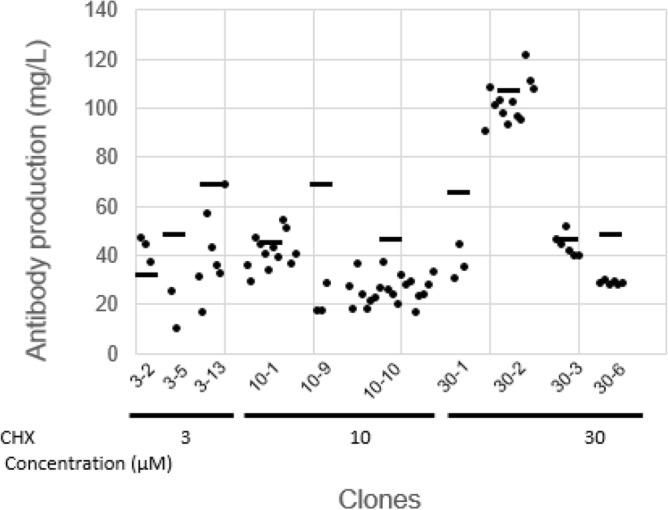
Comparison of clonal cell lines derived from CHX-resistance cell pools. The three or four CHX-resistant cell pools were randomly selected from each selection condition, 3, 10 or 30 μM CHX, and subsequently these cell pools were cloned using a limiting dilution procedure. For a total of 74 clones, 2 to 20 clonal cell line were picked and expanded from each cell pools, the antibody production (closed circle) was measured by protein-A HPLC at the end of 5 days cultivation. Dashed lines represent the average of gene copy number of each parental cell pools in Fig. 3A.

To further characterize cells as clones, we performed a limiting dilution procedure again, for cell pools 3-2, 3-5, 3-13 (from 3 μM CHX selection), 10-1, 10-9, 10-10 (from 10 μM CHX selection) and 30-1, 30-2, 30-3, 30-6 (from 30 μM CHX selection). 2 to 20 clonal cell lines from each pool and, in total, 74 clonal cell lines were isolated, and the antibody production was measured after 5-day suspension cultures (Fig. 4). Clonal cell lines from the 3-2, 3-13, 10-1, 30-2, 30-3 cell pools, we were able to recover clones with productivity comparable to or higher than those of the original cell pools.

**Figure 5:**
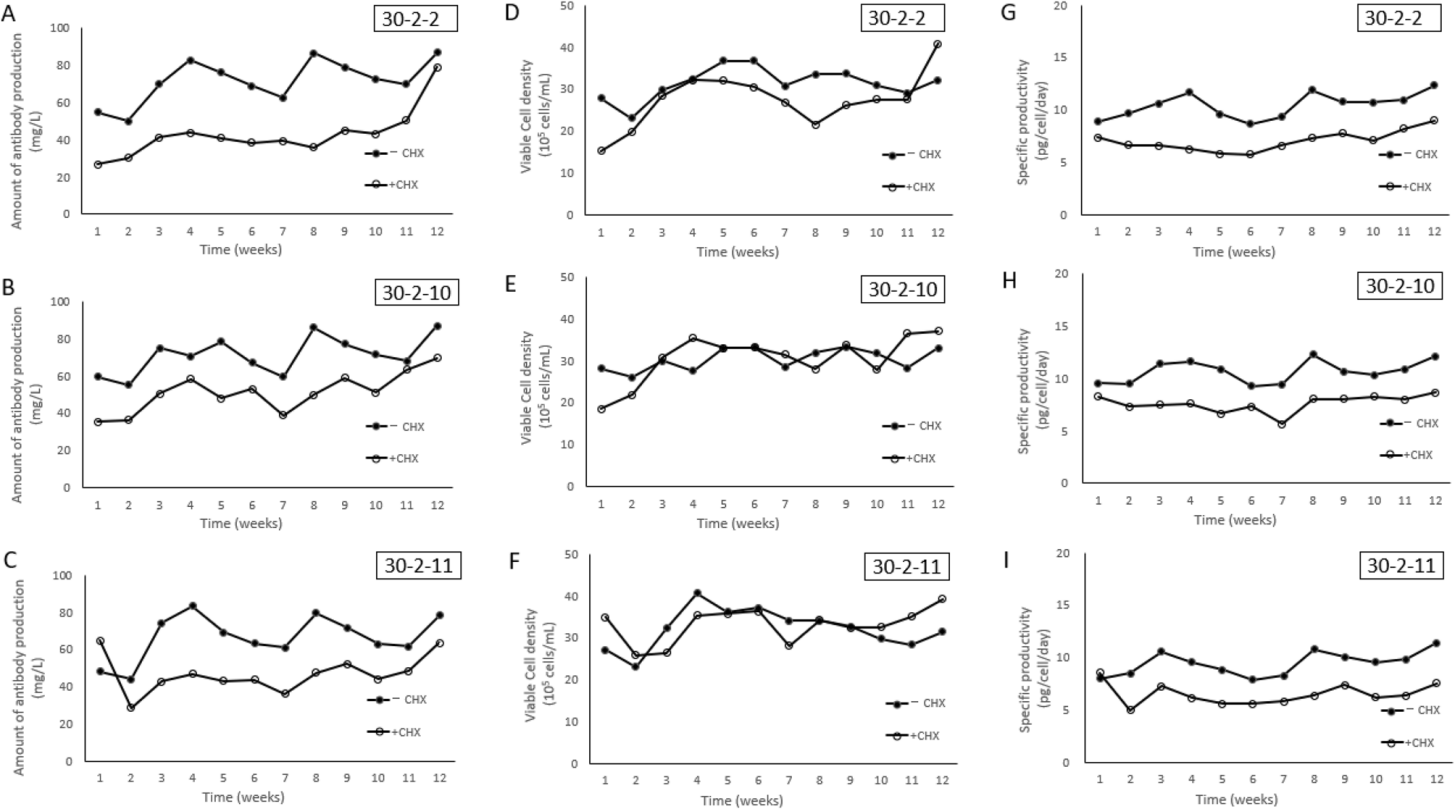
Changes in antibody productivity and cell growth of clonal cell lines 30-2-2, 30-2-10, 30-2-11 in the presence or absence of CHX selection. Changes in antibody productivity (**A**, **B** and **C**), cell growth (**D**, **E** and **F**) and specific antibody productivity (pg/cell/day) (**G**, **H** and **I**) in three clonal cell lines, 30-2-2, 30-2-10, 30-2-11, when long-term cultured in the presence (open circles) or absence (black circles) of CHX selection pressure. Cells were passaged at density of 3 × 10^5^ cells/mL, every 3–4 days and the amount of antibody production (mg/L) by protein-A HPLC and viable cell density (cells/mL) by Vi-CELL XR Cell Viability analyzer were measured once weekly for 12 weeks.

We selected three clonal cell lines, 30-2-2, 30-2-10, 30-2-11 with higher antibody productivities for further studies. To analyze the long-term stability of clonal CHX-resistant cell lines, cells were passaged and seeded at density of 3 × 10^5^ cells/mL every 3–4 days, and were cultivated for 12-weeks in the presence or absence of CHX selection. We measured the antibody productivity and viable cell density every week (Fig. 5). The 30-2-2 line produced 50.2–86.8 mg/L antibody without CHX and 26.8–78.9 mg/L antibody with CHX, the 30-2-10 line produced 55.1–87.1 mg/L antibody without CHX and 35.5–69.6 mg/L antibody with CHX, and the 30-2-11 line produced 44.1–83.3 mg/L antibody without CHX and 28.5–64.7 mg/L antibody with CHX (Fig. 5A,B,C). Specific antibody productivities (pg/cell/day) were rather constant with or without CHX and, in general, higher without CHX (Fig. 5G,H,I). Thus, during this period, both the antibody production and cell viability were rather consistent without or with CHX. It is interesting to note that the antibody production is higher in the absence of CHX.

## Discussion

In the present study, we demonstrated that gene transfer with the Tol2 transposon system in combination with the CHX selection system can generate stable antibody producer cell lines using CHO cells. Three clonal cell lines with the higher antibody productivities isolated in this study showed stable productivity and cell growth up to 12-weeks cultivation period. It has been shown that *N-*glycosylation is important for the efficacy, safety, and pharmacokinetic properties of the antibody^21^. In the present studies, we focused on development of a novel protein production method and the experiments were performed on scales too small to tolerate the glycosylation analysis. We will analyze the quality of antibodies produced using this method in the future study.

The CHO cell has been used as the most popular host cell lines for commercial production of many therapeutic proteins. For commercial purposes, established CHO cells are expected to stably express target proteins with high productivity and acceptable quality for human use. There had been risks of gene silencing with the conventional transfection method. When the transgene was integrated into transcriptionally active region of the host genome, DNA methylation within the integrated transgene or its promoter region was induced by negative positional effect, resulting in silencing of the transgene^5,22^, and, when the transgene was integrated as multicopy, induced heterochromatin formation at the inserted locus and suppressed transgene expression were reported in CHO cells^23^. Further, when the producer cell line was created through the gene amplification method, a decrease in productivity due to loss of transgene copy number has been observed^24,25^. The transposon system can create single copy insertions at multiple loci and should overcome these problems^13^.

Mostly, generating producer cell lines has been carried out by the conventional transfection and gene amplification method^3,4^, and the screening process is laborious and time-consuming since superior lines have to be selected from an enormous number of transfected cell pools. The selection and screening of producer cell lines using adherent cells are carried out serum-based cultivation and then cell lines are adapted to serum-free conditions prior to scale-up. The disadvantages of using serum include safety issues, cost, drawbacks in the purification process of recombinant protein, and animal welfare considerations. Serum is undefined with a high batch-to-batch variability and the process of adaptation to serum-free suspension cultures involves the risk of adverse clonal changes^26^. Therefore, it is desired to establish producer cell lines under serum-free suspension condition that can be directly scaled up to serum-free suspension culture. Our present study demonstrated efficient generation of producer lines using suspension CHO cells under serum-free condition. This was achieved presumably due to high integration efficiencies catalyzed by the Tol2 transposase.

CHX is known as a potent inhibitor of protein synthesis, the resistance gene was constructed by converting the 56th proline to glutamine in yeast ribosomal protein L41^17,18^. In the same manner, it has been demonstrated that the conversion of the 54th proline into glutamine of the human L36a resulted in CHX resistance^19^. However, by our hand, CHX-resistant CHO cells could be obtained under adherent culture condition, but could not be obtained under suspension culture condition by using conventional transfection method (data not shown). In the present study, we showed that CHX-resistant CHO cell pools can be obtained by using the Tol2 transposon system under suspension culture condition with three different CHX concentrations, 3, 10 and 30 μM, presumably due to higher efficiencies of integration of an exogenous gene into the genome. The CHX-resistant CHO cells proliferate well and similarly under suspension culture condition either in the presence of or in the absence of CHX. Interestingly, the protein production from the CHX-resistant CHO cells was higher in the absence of CHX, suggesting that the L36a mutant ribosome can circumvent the inhibitory effect on cell proliferation by CHX, but is still sensitive to that on protein production by CHX.

In conclusion, we provide a new procedure of generating stable cell lines in serum-free suspension CHO cells using Tol2 transposon system, combined with a novel selection marker, CHX-resistance. The stable cell lines established in this study showed constant productivity and cell growth for long-enough cultivation periods for recombinant protein production. We found that the amount of protein production does not proportionally increase accordingly to the gene copy number although there is weak tendency that high producer cell pools could be obtained from cells with higher copy numbers. The top three high producer cell pools described in Fig. 3 had 10 (30-2), 4 (3-15), and 15 (30-8) copies, respectively. Even a cell pool with estimated one gene copy (10-8) can yield protein production levels of about half of the levels of the top three. Thus, the advantage of the use of the Tol2 transposon system does not rely on hitting multi-loci on the genome in the transfected cells, rather may rely on increasing the chance of hitting a putative “high producer locus”, which should be more a transcriptionally active site on the genome. This hypothesis will be examined by analyzing the transposon integration sites more in details in the future.

## Methods

### Cell lines

CHO-K1 cells were obtained from the European Collection of Cell Cultures (ECACC 85051005). CHO-K1 cells^20^ were adapted to suspension culture and maintained in a serum-free proprietary growth medium with 4 mM L-glutamine (Gibco) in shake flasks at 37℃ and 5% CO_2_. Cells were passaged at density of 3 × 10^5^ cells/mL, every 3–4 days. Cell density and viability were measured using the Vi-CELL XR Cell Viability analyzer (Beckman Coulter).

### Plasmid construction

The transposase-expressing vector pCAGGS-T2TP for the transient expression of full-length Tol2 transposase have been described previously^13^. The transposase-donor vector pT2AL200R150G contains the enhanced green fluorescent protein (EGFP) gene flanked by the Tol2 cis-sequences essential for transposition^11^.

The pT2LCHC-CHX plasmid has an expression cassette for the human monoclonal antibody, containing the heavy chain and light chain for a human monoclonal antibody, and CHX resistance gene under the cytomegalovirus (CMV) promoter between Tol2 cis-sequences essential for transposition (Fig. 1). The CHX resistance gene in the expression cassette, in which 54th proline of the human ribosomal protein L36a was substituted with glutamine, was described previously^19^.

### Protein quantification

EGFP-specific fluorescence was measured using BD FACSVerse Flow Cytometer (BD Bioscience) with 488 nm laser. Events (10,000 collected per sample) were gated based on forward scatter/side scatter (FSC/SSC) and data were processed using the FACSuite software (BD Bioscience).

The human antibody concentration in the culture supernatant was measured by protein A affinity HPLC with UV detection (Agilent Technologies). Detection of antibodies was performed at 214 nm with an ultraviolet (UV) detector, and their concentrations were determined from calibration curve that was calculated using standard samples.

### Qualification of integrated transgene copy number

The transgene copy number was measured by quantitative PCR (qPCR) with TaqMan probe using 7900HT Fast Real-time PCR system (Thermo Fisher Scientific). For the detection of transgene copy, the forward primer, reverse primer and FAM-labeled prove for antibody light chain gene were 5’- CCACCACACCCTCCAAACA-3’, 5’-CAGGCGTCAGGCTCAGGTA-3’, and 5’- CAACAACAAGTACGCGGCCAGCAG-3’, respectively. The forward primer, reverse primer, and VIC-labeled probe for furin gene (GenBank accession no. U20436.1) in CHO genome, which served as the internal control to determine absolute gene quantitation of transgene, were 5’-CACAAGTCCCACCTAAAAACTGAA-3’, 5’-CCAGTGCCAGTTTGATTAAAGGA-3’, and 5’-TCTTTCTGGGCATGGTGGCGG-3’, respectively. The genomic DNA was extracted from each cell pools using the DNeasy Blood and Tissue kit (QIAGEN) according to the manufacture’s instruction. PCR was performed with a pre-incubation at 50 °C for 2 min and at 95 °C for 10 min followed by 40 cycles of amplification at 95 °C for 15 s and at 60 °C for 1 min. All samples were tested in duplicate. The 2^−ΔΔCt^ method^27^ was used to analyze the relative copy number from qPCR experiment.

### DNA transfection and generation of recombinant cell pools and clonal cell lines

The CHO cells were suspended in PBS at a density of 1 × 10^7^ cells/mL, and 4 × 10^6^ cells per cuvette were transfected with both 10 μg of donor vector and 5–25 μg of transposase expression vector in circular form by electroporation using Gene Pulser XCell (BioRad Laboratories).

For EGFP expression, the cells were transfected with 10 μg of donor vector pT2AL200R150G and 5, 10, 15 or 25 μg of pCAGGS-T2TP. All conditions were performed in triplicate. Transfected cells were maintained for 5 days with static cultivation, and then cells were transferred to a vented shake flask. Suspension cultivation in shake flask were performed by agitation at 100 rpm. Cells were passaged every 3–4 days on 15 days with inoculation at a density of 3 × 10^5^ cells/mL. For human antibody expression, cells were transfected with 10 μg of pT2LCHC-CHX and 25 μg of pCAGGS-T2TP. The conventional transfection method as a control, cells was transfected with linearized 10 μg of pT2LCHC-CHX without any transposase expression vector. After the transfection, cells in a cuvette were diluted in 20 mL medium and seeded at a density of 4 × 10^4^ cells/well on 96-well plates followed by cultured for 3 days without CHX selection. At 4 days post-transfection, 3, 10 or 30 μg/mL of CHX was added to each plate, followed by culturing for 3 weeks while carried out the medium exchange in the presence of each concentration of CHX in every week. After selection of CHX in 96-well plates, the number of wells in which CHX-resistant colonies were detected was counted as a cell pool. The obtained CHX-resistant cell pools were expanded into 24-well plates and further expanded into 6-well plate while maintained CHX selection. Cultivation in 6-well plates was performed at 90 rpm in suspension culture, and selective medium was replaced every 3–4 days. The CHX-resistant cell pools were randomly selected from each condition of CHX selective pressure (3, 10 or 30 μg/mL) then either subjected to a 5-days batch culture following inoculation at 0.5 × 10^6^ cells/mL into vented shake flask (100 rpm).

Clonal cell lines were recovered from selected cell pools by limiting dilution. Cell pools were then seeded in 384-well plates at a density of 0.15 cells/well in 50 μL of a conditioned medium. After cultivation for about two weeks, individual wells in which colonies formed were expanded sequentially in 96-well plates, 24-well plate, 6-well plate and vented shake flask. Suspension cultivation into 6-well plate and vented shake flask were performed by agitation at 90 rpm and 100 rpm, respectively. The obtained two to twenty of CHX-resistant clonal cell lines from each cell pool were subjected to a 5-days batch culture following inoculation at a density of 0.5 × 10^6^ cells/mL (100 rpm).

To study the stability of the human antibody expression and cell growth over time, the three clonal cell lines were maintained in suspension culture with or without CHX. Cells were passaged every 3–4 days for 12-weeks with inoculation at a density of 3 × 10^5^ cells/mL. The cell number and viability were measured by Trypan Blue exclusion method using Vi-CELL XR (Beckman Coulter), and the human antibody concentration were measured. Specific antibody productivities are given in pg cell^−1^ day^−1^.

## Data Availability

The data collected from this study are available from the corresponding authors on reasonable request.
